# Reversible Cryopreservation of Living Cells Using an Electron Microscopy Cryo-Fixation Method

**DOI:** 10.1371/journal.pone.0164270

**Published:** 2016-10-06

**Authors:** Jan Huebinger, Hong-Mei Han, Markus Grabenbauer

**Affiliations:** Department of Systemic Cell Biology, Max-Planck-Institute of Molecular Physiology, Otto-Hahn-Str. 11, D-44227 Dortmund, Germany; University of California at Berkeley, UNITED STATES

## Abstract

Rapid cooling of aqueous solutions is a useful approach for two important biological applications: (I) cryopreservation of cells and tissues for long-term storage, and (II) cryofixation for ultrastructural investigations by electron and cryo-electron microscopy. Usually, both approaches are very different in methodology. Here we show that a novel, fast and easy to use cryofixation technique called self-pressurized rapid freezing (SPRF) is–after some adaptations–also a useful and versatile technique for cryopreservation. Sealed metal tubes with high thermal diffusivity containing the samples are plunged into liquid cryogen. Internal pressure builds up reducing ice crystal formation and therefore supporting reversible cryopreservation through vitrification of cells. After rapid rewarming of pressurized samples, viability rates of > 90% can be reached, comparable to best-performing of the established rapid cooling devices tested. In addition, the small SPRF tubes allow for space-saving sample storage and the sealed containers prevent contamination from or into the cryogen during freezing, storage, or thawing.

## Introduction

Rapid cooling of aqueous solutions is a powerful tool in life science for at least two important biological and biomedical applications: (I) cryofixation of samples for (ultra-) structural investigations by (cryo-) microscopy, and (II) cryopreservation of living samples for long-time storage.

Most cryopreservation strategies aim to minimize intracellular ice crystallization during cooling. After the discovery of cryoprotectant effects of substances like glycerol [1] or dimethylsulfoxide (DMSO), it had become possible to preserve mammalian cells with slow freezing methods. These methods allow for extracellular ice formation, and cells survive in the unfrozen fraction between the ice crystals [2,3]. However, this approach appeared to be not sufficient to preserve all kind of cells and tissues. Therefore, a rapid cooling approach was developed using high concentrations of cryoprotective agents to completely prevent ice crystal formation [4]. Although the complete suppression of ice crystallization is not necessary as cells tolerate certain small ice crystals [5], the method proved to be highly useful for several cell and tissue types [2,6–8]. Subsequently, cryopreservation protocols have been divided into “slow-freezing” approaches, that allow for the formation of extracellular ice crystals and “vitrification” approaches that seek to prevent any ice formation (for reviews see: [2,9]).

**Fig 1:**
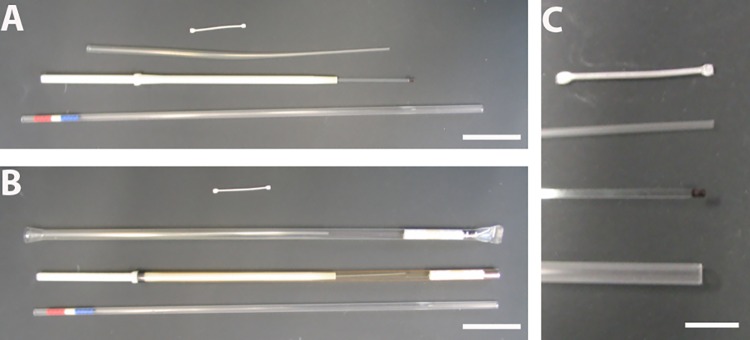
Different devices for cryo-preservation by fast-freezing. **A**: From top to bottom: SPRF-tube, open pulled straw (OPS), cryotop, and mini straw. **B**: The devices are shown with their casing for storage in liquid nitrogen, if available. **C**: Higher magnification of the sample storage area. The sample is pipetted on the black area of the cryotop. All other devices are tubes, in which the sample is sucked into. In OPS and mini straw, the sample is filled in the tip only, whereas the SPRF tube is completely filled. Scale Bars: A, B: 15 mm; C: 5 mm.

Various cryo-protective agents and mixtures of cryo-protectants have been developed aiming to be not cytotoxic in concentrations that suppress ice crystal formation [4,10–15]. Additionally, some attempts were made to improve cooling and warming speed, which allows reducing cryoprotectant concentrations and thereby cytotoxicity [16–20]. Two frequently used cooling and storage devices are the open pulled straw (OPS) and the cryotop (Fig 1).

The OPS is basically a thin plastic straw, which is not sealed to ensure faster cooling [17]. However, the poor thermal conductivity of plastic seems to be the rate-limiting step in cooling with this device [16,21]. The cryotop system is completely open–designed to place a small drop of sample solution containing one or very few oocytes on it. The tip of the cryotop is dipped directly into liquid nitrogen to ensure high cooling rates [8,22]. Both, OPS and cryotop procedures have been proven suitable for survival of cells after vitrification, with advantages for the cryotop system in direct comparison [17,20,22,23]. However, the direct contact of the sample to the cryogen in both devices might lead to cross-contamination of the samples, which is especially problematic if infectious or biosafety level material is stored [2,24–27].

In parallel to developments in cryopreservation, vitrification procedures for cryo-fixation of biological material for subsequent ultrastructural analysis by (cryo-) electron microscopy have been established. In these approaches, the thorough vitrification is mandatory to keep the samples in a ‘close to native’ state, since crystallization of ice displaces biological molecules and alters the ultrastructure [28]. Additionally, the amount of cryoprotectants needs to be minimized, because their presence can as well compromise the ultrastructure severely. Hence, for specimen such as mammalian cells or tissues, technical solutions have been developed for rapid cooling and vitrification. High pressure freezing (HPF) has been established as the standard method for cryofixation for electron microscopy of bulk specimen. In the HPF device, a pressure of app. 2100 bar supports the vitrification of the specimen during fast cooling [29–31]. It has been supposed that application of high pressure equals a 20% reduction of cryoprotectants [30], or ensures a ten times thicker vitrification depth compared to ambient pressure [32]. Such rapid cooling devices might be beneficial for cryopreservation, but mammalian cells or tissue do not survive established HPF procedures and subsequent warming, probably due to the lack of appropriate warming methods (S1 Fig).

Recently, a new cryofixation method–named self-pressurized rapid freezing (SPRF)–has been developed, that utilizes rapid freezing of a sample placed in a confined volume of a tightly closed metal tube [33]. In SPRF, cells are plunge-frozen in metal tubes that have superior thermal diffusivity (aluminum 84 mm^2^/s; copper 117 mm^2^/s), compared to plastic (0.1 mm^2^/s) vitrification devices [16]. The expansion of water and hexagonal ice during cooling causes increased pressure inside the tube and thus supports vitrification of the remaining sample similar to the LeChatelier principle. In subsequent studies, it has been proven by cryo-electron microscopy, that a modified procedure indeed enables vitrification of biological material like mammalian cells, and to a certain extend their survival [5,34,35]. The combined beneficial effects of high thermal diffusivity and pressure in the confined volume might also enable an efficient rewarming process. This indicates SPRF as promising tool for reversible cryopreservation of living cells.

We further optimized the SPRF procedure to become a useful method for cryopreservation, reaching viability rates comparable to established open devices, with the advantage of being sealed and space saving in the liquid nitrogen storage.

## Material & Methods

### Culturing of bacteria, yeast, mammalian cells and worms

*Escherichia coli K12 (E*. *coli)* bacteria were grown over night in 5 mL LB medium at 37°C and 200 rpm. *Saccharomyces cerevisiae (S*. *cerevisiae)* yeast was cultured on yeast extract peptone dextrose (YPD) agar plates. Before the experiments, yeast was incubated over night in 5 mL liquid YPD at 30°C and 200 rpm. HeLa (ATCC No. CCL-185), Cos-7 (ATCC No. CRL-1651), and MDCK (ATCC No. CCL-34) cells were grown in Dulbecco’s modified Eagle’s serum (DMEM), supplemented with 10% fetal calf serum (FCS), 2 mM L-glutamine, and 1% non-essential amino acids (NEAA). Mammalian cells were grown in 250 mL Falcon tissue culture flasks at 37°C with 5% CO_2_. As first step in cryopreservation experiments, the mammalian cells were trypsinized, washed, and taken up in suspension in the indicated media. The cell concentration was always adjusted to 2.5x10^7^ cells/mL. *Caenorhabditis elegans* worms *(C*. *elegans)* were cultured on plates seeded with *Escherichia coli OP 50 (E*. *coli)* bacteria. Before the experiments, the worms were washed off the plates with phosphate-buffered saline (PBS).

### Composition of cryoprotective media

EAFS is a mixture of the cryoprotective agents ethylene glycol, acetamide, Ficoll and sucrose. It was originally developed for cryopreservation of oocytes [13] and is still used for this purpose [8,36]. We followed the published protocols for EAFS 10/10, where 10% ethylene glycol (Serva, Heidelberg, Germany) and 10.7% acetamide (Acros Organics, Geel, Belgium) were dissolved in a solution of 30% (w/v) Ficoll PM 70 (GE Healthcare, München, Germany) and 0,5 M Sucrose (Serva, Heidelberg, Germany) in PB1 medium. PB1 medium is PBS supplemented with 3 g/L BSA, 1 g/L glucose and 0.036 g/L sodium pyruvate.

A mixture of DMSO, ethylene glycol and sucrose has been used recently for vitrification approaches in cryopreservation [37]. We use here 15% DMSO, 15% ethylene glycol and 0,5 M sucrose (all Serva, Heidelberg, Germany) dissolved in PBS containing 20% fetal bovine serum (FBS)[37]. In analogy to EAFS, which is abbreviated using the first characters of its cryoprotective agents, we use DES as acronym for this medium.

Dextran is frequently used as cryo-protectant for cryo-electron microscopy of vitrified sections (CEMOVIS) experiments, usually in concentrations of 20–30% (w/v)[38,39]. We used dextran40 (40 kD average molecular mass, Sigma-Aldrich, Taufkirchen, Germany) as 30% solution in PBS. In some indicated experiments, a mixture of ethylene glycol and dextran was used, where we suspended 10% ethylene glycol (Serva, Heidelberg, Germany) in a solution of 30% dextran (MW 35kDa; Sigma-Aldrich GmbH, Taufkirchen, Germany) in PBS.

### Self-pressurized rapid freezing (SPRF)

Mammalian cell suspensions were diluted to densities of 2.5x10^7^ cells/mL. *S*. *cerevisiae* from overnight culture were centrifuged and resuspended in 50 μL of the indicated media. *C*. *elegans* were mixed with yeast paste. *E*. *coli* from overnight cultures were centrifuged and resuspended in 200 μl PBS. Suspensions in indicated media were taken up into metal tubes as described [34,35]. We used aluminum tubes (300 μm inner and 600 μm outer diameter; Goodfellow GmbH, Bad Nauheim, Germany) and silver tubes (300 μm inner and 600 μm outer diameter; Heimerle & Meule GmbH, Pforzheim, Germany) cut to a length of 15 mm. First, tubes were inserted into a disposable pipette tip mounted on a 0.5–10 μL micropipette. Next, the micropipette volume was set to 3–4 μL in excess of the tube capacity (about 1 μL) to allow for overfilling of the capillary. Then the open end of the capillary was inserted into the specimen suspension and the suspension was very carefully drawn into the tube, in order to prevent formation of air bubbles inside the capillary. Afterwards, the tubes filled with the sample were sealed at both ends using pliers with flat jaws. Tubes were mounted on fine tweezers and plunged horizontally into liquid ethane cooled by liquid nitrogen (−196°C) using a custom-made plunge-freezer. For some indicated experiments, tubes were plunged into liquid nitrogen by hand.

For rapid re-warming, the tubes containing mammalian cells were transferred as quickly as possible from liquid nitrogen to a directly adjacent water bath set to 37°C. *S*. *cerevisiae* were warmed in a tube containing 200 μL pre-warmed YPD (30°C). Afterwards, one end of the tube was cut open with scissors; the tube was mounted on a pipette tip (0.1–10 μL) whereby the closed end was sticking out of the pipet tip. The pipette tip was mounted on a micropipette, the closed end of the tube was cut open and the solution was pipetted in 200 μL cell culture medium in an 8-well glass-bottom cell culture dish (Thermo Fisher Scientific, Rochester, USA).

### Rapid cooling by the cryotop method

The cryotop (Gynemed Medizinprodukte GmbH, Lensahn, Germany) consists of a thin plastic tip, on which a small amount of cell suspension or single oocytes can be loaded, with a relatively large handle (Fig 1). We loaded 0.5 μL of cell suspension on this tip. Using smaller volumes or less cells impeded quantification of viability rates. Larger volumes are difficult to load on the tip and would reduce cooling or warming speed. For rapid cooling, the device was held parallel above the liquid nitrogen surface before the tip was rotated into the liquid nitrogen. This technique was shown to foster faster cooling than just dipping the device in the liquid nitrogen [16]. For thawing, the tip of the device was transferred as fast as possible from the liquid nitrogen to pre-warmed cell culture medium at 37°C. Cell suspensions from two or three tips were pooled in one well of an 8-well glass-bottom cell culture dish (Thermo Fisher Scientific, Rochester, USA) and cultured in 200 μL cell culture medium therein.

### Rapid cooling in open pulled straws (OPS)

To load the OPS (Vajta embryology consulting, RVT, Australia), a small drop (2 μL) of cell suspension was placed on a petri dish. The drop was taken up into the OPS by capillary forces. For freezing, the tip of the OPS was brought into liquid nitrogen in the same way as the cryotop, to ensure the fastest possible cooling. The device was hold parallel above the surface of the liquid nitrogen and the tip was rotated in the liquid nitrogen. The OPS was warmed in a 0.5 μL tube (Eppendorf AG, Hamburg, Germany) containing 200 μL cell culture medium at 37°C. This ensured fast and uniform warming by immersing the whole cell suspension into the medium. Finally, the cell suspension was transferred to 8-well glass-bottom cell culture dishes (Thermo Fisher Scientific, Rochester, USA).

### Rapid cooling in mini straws

Mini straws of 0.25 mL (MTG Medical Technology Vertriebs-GmbH, Bruckberg, Germany) were filled with 146 μL cell culture medium. Separated by an air bubble, 4 μL of cell suspension were filled afterwards and the straws were heat-sealed. This type of filling is adapted from a technique of filling these straws for in-straw rehydration [18]. Smaller volumes than 4 μL do not fill the whole diameter of the straw. Thus, they would be difficult to be kept in position during handling before rapid cooling and during mixing with the cell culture medium. After thawing in a 37°C water bath, the straws were cut open and emptied into one well of a 24-well-plate containing 250 μL pre-warmed cell culture medium.

### High-pressure freezing

*S*. *cerevisiae* from overnight cultures were centrifuged and re-suspended in 100 μL medium. These cell suspensions were pipetted into aluminum carriers with diameters of 3 mm and cavities of 300 μm (Engineering Office M. Wohlwend GmbH, Sennwald, Switzerland). The aluminum carrier was covered with the flat bottom of another carrier and cooled with liquid nitrogen at 2000 bar in a high-pressure freezing machine (HPM 100; Leica Microsystems, Vienna, Austria). Suspensions were subsequently warmed in 200 μL YPD medium at 30°C.

### Cell viability test

The viability of mammalian cells after cooling was quantified by assessing the proportion of cells that were able to re-adhere. We chose this robust quantification method since all kinds of cell deaths are considered, not just direct membrane ruptures. After seeding in the cell culture dishes, cells were cultured at 37°C and 5% CO_2_. Control cells always adhered within three hours. Cells not adhering within three hours after re-seeding did also not adhere later on, as observed microscopically. Because of this observation, adherence was always quantified after six hours. Therefore, the supernatant was removed and cell concentration was determined using a hemocytometer. This reflects the fraction of dead cells not able to re-adhere. Adherent cells were washed once in PBS, detached by trypsinization and taken up into a solution of fresh medium. The concentration of these cells was also determined using a hemocytometer.

*S*. *cerevisiae* were cultured in YPD for 20 min at 30°C and 200 rpm, before quantification of viability by propidium iodide (PI) staining. Subsequently, the suspension was mixed 1:1 with a PI solution (12 μg/mL in PBS), to stain dead cells. The PI-staining was analyzed in a LSR2 FACS apparatus using the 488 nm laserline and a 610/20 nm band pass filter (BD Biosciences, Heidelberg, Germany). To quantify viability by cell growth, *S*. *cerevisiae* were cultured in YPD for 24 h at 30°C and 200 rpm and the optical density was measured at 600 nm. It was confirmed microscopically that *S*. *cerevisiae* were growing without any contamination.

### Freeze substitution

Freeze substitution was performed as described before [34], following a protocol to enhance contrast by addition of small quantities of water [40]. First, tubes containing samples were cut in pieces of 1–2 mm length under liquid nitrogen using the Leica cryotools (Leica Microsystems, Vienna, Austria). Then, samples were transferred as fast as possible from liquid nitrogen into pre-cooled substitution medium consisting of acetone containing 0.1% osmium tetroxide, 0.1% uranyl acetate and 5% water, pre-cooled to -90°C in an automatic freeze substitution apparatus (AFS2; Leica Microsystems, Vienna, Austria). After warming from -90°C to 0°C over at least 16 h using an exponential warming protocol, samples were kept at room temperature for 1 h. Afterwards, capillary tube segments were removed and samples were pelleted in an Eppendorf tube. Before embedding in Epon 812, samples were washed twice with pure acetone for 1 h, incubated for 2 h in an 1:1 mixture of Epon/acetone, another 2 h in pure Epon at room temperature, and again 2 h in pure Epon at 37°C. Finally, Epon was polymerized at 60°C for at least 36 h.

#### Vitreous sectioning

Vitreous sectioning was performed as described before [34]. SPRF tubes containing the vitrified sample were fixed in a pre-cooled (−145°C) EM FC6 cryo-ultramicrotome (Leica Microsystems, Vienna). The sample was trimmed to a rectangular shaped block of 70–100 μm base and app. 50 μm height using a 20° cryo trimming diamond knife (CT1303; Diatome, Biel, Switzerland). A 25° cryo diamond knife (MT9876; Diatome, Biel, Switzerland) with a clearance angle of 6° was used to get ribbons of cryo-sections at a nominal cutting feed of 50 nm and at cutting speeds of 10 mm/s. Ribbons of cryo-sections were attached to pre-cooled 600 mesh EM grids with an eyelash using electrostatic charging with the Crion™ (Leica Microsystems, Vienna) [41].

### Electron and cryo-electron microscopy

Electron microscopy was performed as described before [34]. Grids with the vitreous sections were placed in a pre-cooled cryo-specimen holder (Gatan 626-DH; Warrendale, PA, USA) and transferred into an electron microscope (JEOL JEM-1400; JEOL Germany, Eching, Germany). The accelerating voltage was set to 120 kV and microscopy was performed at −178°C. The ice state in the sections was confirmed by electron diffraction. The micrographs were digitally recorded on a 2K×2K or 4K × 4K CCD camera (F-214FS or F-416; TVIPS, Gauting, Germany). The electron dose on the specimen was kept between 700–1500 e^-^/nm^2^. Plastic sections were analyzed accordingly in the same microscope without the precautions needed for vitreous sections.

## Results

### Vitrification and pressure in SPRF tubes

**Fig 2:**
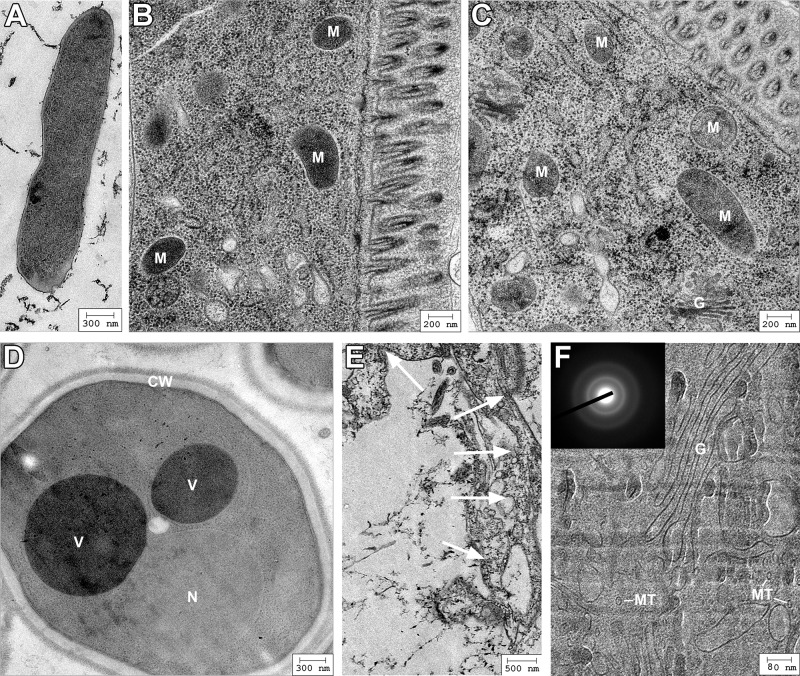
Ultrastructure of different organisms and cultured cells after SPRF. **A-E**: Electron micrographs after SPRF fixation without cryoprotectant, subsequent freeze substitution, and ultrathin sectioning of E. coli (A), C. elegans (B,C), S. cerevisiae (D), and Cos7 cells (E). **F**: Representative cryo-electron microscopy of vitrified section of a mammalian cell after SPRF in the presence of 30% dextran. Diffraction pattern of the sample is shown in the insert verifying that the sample is vitrified. Note acceptable ultrastructural preservation in A-D and E, with several recognizable cellular components: mitochondria (M), Golgi fields (G), vacuoles (V), nucleus (N), cell wall (CW), microtubules (MT). The mammalian cell frozen without cryoprotectant in E is severely damaged, its outer shape is not discernable and membranes are highly disordered (white arrows).

**Fig 3:**
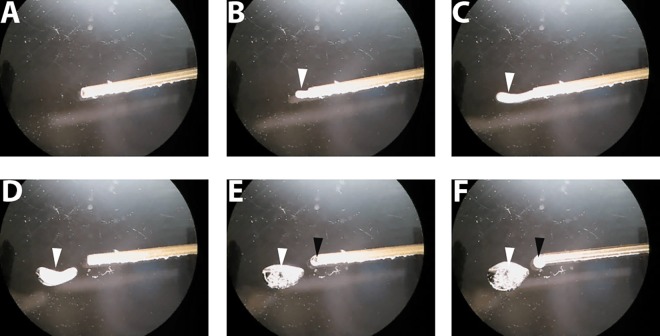
SPRF tube during re-warming under a stereomicroscope. After SPRF, an aluminum tube filled with EAFS medium was cut open under liquid nitrogen and observed under a stereomicroscope during re-warming. Shown are images before ice expansion (**A**), and 130 ms (**B**), 195 ms (**C**), 260 ms (**D**), 520 ms, and (**E**) 1495 ms after first visible expansion occurs (see also S1 Video). Ice expands out of the tube (white arrowheads in B and C) and melts subsequently (white arrowheads D-F). Afterwards liquid drops out of the tube (black arrowheads in E and F).

In the first description of self-pressurized rapid freezing (SPRF), it was assumed that the pressure inside SPRF tubes reaches up to app. 2000 bar, thereby ensuring vitrification even without the use of cryoprotective agents [33]. Using cryo-EM, it was shown subsequently that cell suspensions–without cryoprotective agents–do not vitrify through SPRF [34,42]. Astonishingly, the ultrastructure of many microorganisms is still reasonably well preserved in these tubes and can be evaluated after freeze substitution [33–35], but mammalian cells are mostly destroyed unless a cryoprotective agent like dextran is used (Fig 2). Due to their larger volume and higher water content, mammalian cells might be more sensitive to freezing damage. However, the actual pressure inside of the confined volume of the small metal tubes could not be measured directly during freezing and remains unknown. By stereomicroscopy, we visualized the pressure-driven expansion of ice that occurs upon thawing SPRF tubes (Fig 3, S1 Video). This shows qualitatively the increase of pressure within the tubes. Furthermore, this pressure is also generated during warming/thawing, thereby potentially suppressing ice crystal formation during this critical process.

### Cellular survival after SPRF

**Fig 4:**
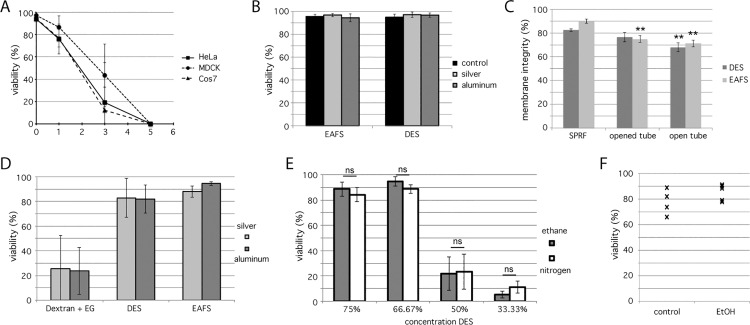
Viability of mammalian cells in SPRF tubes. **A:** Viability of MDCK (○), HeLa (■) and Cos7 (△) cells in PBS after indicated presence in copper SPRF tubes at room temperature, quantified by their ability to re-adhere (n = 5). **B:** Viability of HeLa cells suspended in cryoprotectants (EAFS and DES) after 60 sec in sliver or aluminum tubes at room temperature quantified by their ability to re-adhere. Cells suspended in the according media served as controls (n = 5). **C:** Membrane integrity of HeLa cells suspended in cryoprotectants (EAFS and DES) assessed by PI-staining after cryopreservation in sealed SPRF tubes (SPRF), in tubes that were sealed, plunge-frozen, opened under liquid nitrogen and then thawed (opened tubes) and tubes that were not sealed before plunge-freezing (open tubes). All tubes were thawed in air at room temperature instead of a water bath at 37°C (n = 10); **D:** HeLa cells suspended in indicated CPA mixtures were frozen in aluminum or silver tubes. Their viability was quantified by their ability to re-adhere after thawing. Additionally to the cryoprotectant mixtures DES and EAFS, which lead to high viability rates, a mixture of 27% dextran and 10% ethylene glycol (Dextran + EG) was used (n = 6). **E:** HeLa cells suspended in different dilutions of DES medium were cooled in aluminum tubes either by plunging into liquid ethane with the help of a plunge-freezer (grey bars) or by directly plunging into liquid nitrogen by hand (white bars). Their viability was quantified by their ability to re-adhere after thawing (n = 5). **F:** A suspension of HeLa cells in PBS was filled in SPRF-tubes. The tubes were immersed in a 70% ethanol bath for 30 s, and re-cultured afterwards for quantification of viability. Controls were treated the same, except for immersion into ethanol. All data are represented as mean ± s. d.; significance was tested using student’s t-test; **: p<0.01; ns: p>0.05.

Immortalized mammalian cells are a well-suited model system to test viability rates after cryopreservation. Culturing in large quantities allows for robust quantification of their survival. Unlike budding yeast cells (*S*. *cerevisiae*), which survive SPRF even without cryoprotectants, mammalian cells are sensitive to freezing damage upon suboptimal freezing/thawing conditions (Fig 2 and S2 Fig). The copper tubes as used in the original publication of SPRF [33] are very poisonous to cells. Already the presence of cells in the tubes for a few minutes without freezing is lethal (Fig 4A). This fatal effect is even enhanced in the presence of dextran as cryoprotectant, which is already lethal after 60 sec [34]. Aluminum tubes, however, have been shown to be biocompatible, also in the presence of dextran [5,34] or mixtures of cryoprotective agents as used in this study (Fig 4B). Similar to aluminum, silver as tube wall material shows no poisonous effects on HeLa cells in these media (Fig 4B).

Cells in PBS frozen without cryoprotectants in SPRF tubes are completely destroyed due to severe ice crystal formation (Fig 2E). But also cells successfully vitrified using dextran as cryoprotectant (Fig 2F), die after cryopreservation [5]. Therefore, we studied and compared cryopreservation properties of SPRF tubes using approved cryoprotective media. A well-established cryoprotective mixture is EAFS (a mixture of ethylene glycol, acetamide, Ficoll, and sucrose; see Material and Methods), which is still used in recent studies as cryoprotectant for oocytes [8,13,43]. The second cryoprotective mixture used here is made of DMSO, ethylene glycol and sucrose [37]. In analogy to EAFS we abbreviate this medium “DES” by using the first letters of its cryoprotective compounds (see Materials and Methods for exact composition of cryoprotective media). Preserving cells in these media using SPRF yielded in high viability rates of app. 90% (EAFS) and 80% (DES) (Fig 4C and 4D). To evaluate, if the pressure inside the tube has an influence on the survival, we preserved cells in closed tubes, open tubes, or cut closed tubes open in liquid nitrogen after cooling. To simulate suboptimal warming conditions, these samples were thawed in air. Both, cooling open tubes and opening closed tubes before warming lead to a significant decrease in membrane integrity (Fig 4C), indicating that the tubes inherent pressure indeed supports higher viability rates, especially under suboptimal conditions.

To improve freezing properties, we used tubes made of silver instead of aluminum. Silver has a nearly 2-fold higher thermal diffusivity (165 mm^2^/s) compared to aluminum (84 mm^2^/s). However, viability rates did not improve by freezing and thawing in silver tubes using different cryoprotective media, including one medium that allows only for suboptimal survival (Fig 4D). It has been shown recently, that the cooling rate is at some point limited by the geometry of the sample and cannot be further improved by increased thermal diffusivity of tube wall materials [16]. To test, if we already reached this limit, we reduced the cooling rate by plunging aluminum tubes directly into liquid nitrogen by hand, instead of using a plunge freezer and liquid ethane as cryogen, which is the usual practice for SPRF. Plunged directly into liquid nitrogen, a layer of gaseous nitrogen forms around the sample–the so-called Leidenfrost phenomenon. This leads to thermal insulation and therefore a lower cooling rate. However, this direct plunging did not lead to lower viability rates (Fig 4E), indicating that the cooling rate already reached an optimum, as defined by the geometry of the sample.

### Comparison between SPRF, OPS, cryotop and mini straw

Comparing the design of SPRF vitrification devices to those typically used for cryopreservation like OPS or cryotop, we find two major practical advantages. First, SPRF tubes are by far the smallest vitrification devices, compared to established ones (Fig 1). However, sample volumina inside these devices are quite similar with slightly increasing volumes from cryotop < SPRF < OPS < mini straw (Table 1). Therefore, app. 100x more sample volumes can be stored in a liquid nitrogen tank using SPRF tubes versus OPS. Compared to the cryotop system this factor is even much higher.

The second advantage of SPRF tubes is their closed configuration. Apart from internal pressure built up, this has two positive effects. First, the risk of contamination in liquid nitrogen is minimized. To remove potential contamination on the tubes outer side, they may be washed and sterilized after thawing. Second, tightly closed tubes could be cooled, stored or warmed in potentially poisonous cryogens or liquids. By warming samples in substances with improved properties like higher thermal conductivity or better thermal diffusivity, the warming velocity could be further optimized. Faster cooling did not show a positive effect on survival (Fig 4D and 4E), but faster warming might be beneficial.

**Table 1:** Comparison of sample volumes vs. storage space of cryopreservation devices. Shown is the typical sample size in different vitrification devices compared to the minimum storage space as needed in liquid nitrogen tanks. The storage space is calculated from the outer dimensions of the devices, for OPS and cryotop the casing is taken into account (compare Fig 1).

	Sample size (μL)	Storage space (μL)	Sample/Storage
**mini straw**	4*	350	1.1%
**OPS**	~2	810	0.25%
**cryotop**	0,1–0,5	790	0.013–0.06%
**SPRF**	1*	4	25%

* volume used in this study, potentially larger volumes may be used.

To test proper sealing, we filled SPRF tubes with a sample containing HeLa cells, sealed the tubes tightly and placed them into a bath of 70% ethanol, which is usually lethal to HeLa cells. After bathing for 30 sec in ethanol, the tubes were washed, opened, and the cells were further cultured. No significant decrease in viability could be measured (Fig 4F). Proper and efficient sealing of the tubes was shown by the fact, that even small amphipathic molecules like ethanol did not reach the tubes interior.

### Measurement of viability rates in different rapid-freezing devices

**Fig 5:**
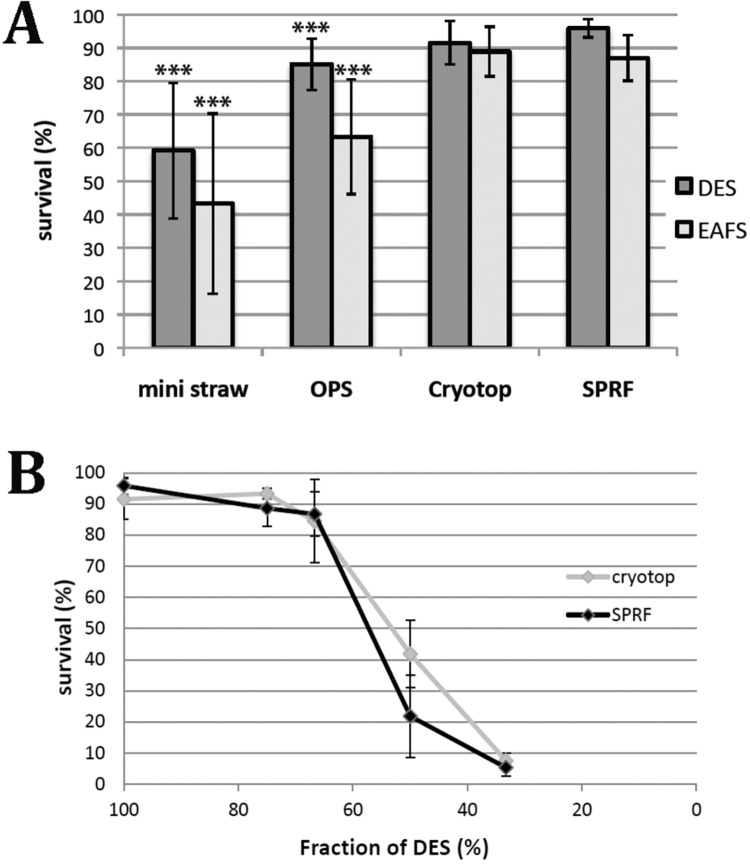
Comparison of viability rates of HeLa cells in mini straws, OPS, cryotop and SPRF tubes. Viability of HeLa cells determined by their ability to re-adhere. **A** HeLa cells suspended in DES (dark grey) or EAFS (light grey) have been cryopreserved using different vitrification devices: mini straw, OPS, cryotop, and SPRF. (n = 12); *** significantly different to SPRF in the same medium (p<0,001 using student’s t-test). **B** HeLa cells were suspended in different dilutions of DES, indicated on the x-axis. They were cryopreserved using SPRF or cryotop (n = 5). Data is represented as mean ± s. d.

By comparing survival of HeLa cells in EAFS and DES media after cryopreservation with different devices, we found similar high viability rates using SPRF tubes or the cryotop method, whereas the viability rates using OPS or mini straws were significantly lower (Fig 5A). HeLa cells reached around 90% viability rates in EAFS or DES media after rapid cooling in SPRF tubes or the cryotop method. This impedes determining clear differences, since the dynamic range of viability rates is limited by the fact that survival cannot exceed 100%. Additionally, the maximum viability rate of 100% is hardly reached as a small proportion of cells might already get lethally damaged without freezing, just during several preparation steps like trypsinization or filling and draining of the vitrification devices. To determine the differences between devices using a higher dynamic range, we diluted the DES medium to approach lower viability rates comparing SPRF and cryotop. Yet, even at these suboptimal conditions no significant difference between SPRF tubes and the cryotop system was detectable (Fig 5B).

## Discussion

### Conceptual implications for cryopreservation

Two factors are generally considered important for the success of cryopreservation: the composition of the cryoprotective media, and the speed of temperature change i.e. cooling or warming. Both factors can be varied to minimize ice crystal formation in cryopreserved cells. Supplementing media with cryoprotective agents is toxic or poisonous to cells, if their concentrations exceed certain levels [2,9]. Therefore, increasing cooling and warming speed to minimize cryoprotectant concentration is a promising approach [2,16,17,19]. In advanced preparation techniques for electron and cryo-electron microscopy, the cooling speed during cryo-fixation has been optimized and is additionally supported by simultaneous application of high-pressure to prevent ice crystal formation enabling the successful vitrification of small samples [28,30,32,33,44]. However, these cryofixation methods were invented for imaging purposes, not for the cryopreservation of cells or tissues, as survival of the sample during warming was not required. Yet, the recently developed cryo-fixation method SPRF allows for cooling down and warming up by suppressing ice crystallization using a confined volume instead of using external pressure as applied in HPF machines. Isochoric (constant volume) cryopreservation has been theoretically proposed earlier [45]. In a pressure vessel that has been demonstrated to enable truly isochoric cooling [46], it has been shown very recently that *C*. *elegans* can survive subfreezing temperatures of –2°C without any loss in viability [47]. The process of SPRF cryopreservation reaches far deeper temperatures, but might not be truly isochoric, as faint volume changes of the pressurized tubes due to wall material elasticity could occur. However, we found that the combination of rapid freezing and using a confined volume in SPRF leads–after significant changes and optimization of the original procedure–to very high survival rates for mammalian/human cells after storage at –196°C. During cooling, SPRF has recently been shown to supress ice crystal formation in the sample and in particular inside cells, depending on the used cryoprotectant [34]. On the other hand, the warming process might in general offer more options to optimize the success of cryopreservation than the cooling process [5,36]. The interesting observation of ice expansion out of the opened tube during re-warming (Fig 3 and S1 Video) shows that the sample itself generates intrinsic pressure during this critical phase of re-warming. Consequently, SPRF allows for very rapid cooling as well as rapid warming, while the elevated pressure level in the confined volume minimizes ice crystallization in both cases.

### Applicability of SPRF for cryopreservation

In agreement with these theoretical considerations, we found experimentally that viability rates after SPRF cryopreservation equal the ones obtained with the cryotop method, which was in our hands the best of the established cryo-cooling methods. However, the volume of cryopreserved cell suspension per device is larger in the SPRF tubes than in the cryotop. So far, faster cooling rates in cryopreservation were mainly achieved by minimizing the sample volume. Consequently, the cryotop–which use the smallest sample volume–yield highest survival rates among the established cryo-preservation devices (Fig 5A)[17,20,22,23]. But this limits the applicability to small samples and low amounts of cells. Cryopreservation by SPRF allows larger volumes to reach high survival rates comparable to cryotop. This effect is most likely obtained through suppression of ice formation by high thermal diffusivity in combination with cooling and warming in confined volumes.

An additional property of SPRF tubes being smaller than established devices is their increased effective storage space in liquid nitrogen, app. 1000-fold higher compared to the cryotop. The possibilities to increase this ratio even further were not explored in this study. For example, due to the beneficial effects of the high thermal diffusivity in combination with the confined volume used in SPRF, even larger volumes might get cryopreserved with the same efficiency in elongated and/or thinner walled tubes. This is in contrast to most cryopreservation strategies, which aim to minimize volumes for better heat transfer out of the sample [20].

Compared to OPS and cryotop methods, SPRF tubes have the advantage to be completely and tightly sealed devices and thereby preventing the risk of contaminations, which is a general problem during cryopreservation and highly important using dangerous biological safety and/or very sensitive material [2,24–27]. In addition, the closed container geometry enables the usage of potentially poisonous liquids for sample warming such as mercury, which has much higher thermal conductivity than water.

Finally, SPRF tubes provide by their handling simplicity the option to perform cryo-preservation outside the laboratory. The basic procedure can be operated even without electricity, allowing to collect living samples of smaller organisms or their parts at extreme habitats and environments, for example at expeditions. As tightly closed devices, SPRF tubes are suitable containers for potentially dangerous organisms or samples that can be collected alive and further cultured in the laboratory and/or directly used for (electron-) microscopy or processed for subsequent biomedical analyses.

For all these reasons, we think that SPRF is a useful method for cryopreservation, and further exploration of its properties seems appropriate. The main advantages of SPRF are especially interesting for large scale bio-banking, where storage space becomes an important issue, and for storage of clinical or biosafety material, where contamination of or from the sample has to be strictly avoided.

## Supporting Information

S1 Fig Viability of HeLa cells after high pressure freezing. HeLa cells were subjected to high pressure freezing in either PBS or PBS supplemented with 30% dextran. They were subsequently thawed in 37°C warm cell culture medium and their viability was assessed by their ability to re-adhere after 6 h of culturing. As a control, unfrozen cells from the same samples were cultured. Data are represented as mean ± s.d.; n = 5–8.
(TIF)

S2 Fig Viability of *S*. *cerevisiae* after different freezing procedures. **A:** Viability of *S*. *cerevisiae* after different freezing procedures, quantified by PI staining. *S*. *cerevisiae* were diluted in YPD-medium, 15% glycerol in YPD (Gly), PBS or 30% dextran in PBS (Dex). They were subjected to conventional freezing in cryo-vials (vial), high-pressure freezing or self-pressurized rapid freezing (SPRF). After subsequent thawing integrity of yeast cells was evaluated by PI-staining. Data is represented as mean ± s.d.; n = 5. **B:** Viability of *S*. *cerevisiae* after SPRF in PBS without cryoprotection, quantified by measuring the optical density at 600 nm after 24 h of growth in YPD medium at 30°C and 200 rpm. Control: *S*. *cerevisiae* from corresponding samples filled in SPRF tubes but not frozen. Data is normalized to the corresponding controls. Black marks are single experiments; in gray mean ± s.d. are represented; n = 5
(TIF)

S1 Video Re-warming of SPRF tube. SPRF aluminum tube filled with EAFS medium was cut open under liquid nitrogen after SPRF. The tube was observed by stereomicroscopy during re-warming.
(MOV)

## References

[1] Polge C, Smith AU, Parkes AS. Revival of spermatozoa after vitrification and dehydration at low temperatures. Nature. 1949;164: 666 10.1038/164666a0 18143360

[2] Pegg DE. Principles of cryopreservation. Methods Mol Biol. 2015;1257: 3–19. 10.1007/978-1-4939-2193-5_1 25428001

[3] Mazur P. Freezing of living cells: mechanisms and implications. Am J Physiol. 1984;247: C125—42. 6383068 10.1152/ajpcell.1984.247.3.C125

[4] Rall WF, Fahy GM. Ice-free cryopreservation of mouse embryos at -196 degrees C by vitrification. Nature. 1985;313: 573–575. 10.1038/313573a0 3969158

[5] Huebinger J, Han H-M, Hofnagel O, Vetter IR, Bastiaens PIH, Grabenbauer M. Direct Measurement of Water States in Cryopreserved Cells Reveals Tolerance toward Ice Crystallization. Biophys J. 2016;110: 840–9. 10.1016/j.bpj.2015.09.029 26541066 PMC4775837

[6] Berthelot F, Martinat-Botté F, Locatelli A, Perreau C, Terqui M. Piglets born after vitrification of embryos using the open pulled straw method. Cryobiology. 2000;41: 116–124. 10.1006/cryo.2000.2273 11034790

[7] Edgar DH, Gook DA. A critical appraisal of cryopreservation (slow cooling versus vitrification) of human oocytes and embryos. Hum Reprod Update. 2012;18: 536–54. 10.1093/humupd/dms016 22537859

[8] Seki S, Mazur P. Ultra-rapid warming yields high survival of mouse oocytes cooled to -196°c in dilutions of a standard vitrification solution. Baltz JM, editor. PLoS One. Public Library of Science; 2012;7: e36058 10.1371/journal.pone.0036058 22558325 PMC3338624

[9] Wowk B. Thermodynamic aspects of vitrification. Cryobiology. 2010;60: 11–22. 10.1016/j.cryobiol.2009.05.007 19538955

[10] Cha SK, Kim BY, Kim MK, Kim YS, Lee WS, Yoon TK, et al Effects of various combinations of cryoprotectants and cooling speed on the survival and further development of mouse oocytes after vitrification. Clin Exp Reprod Med. 2011;38: 24–30. 10.5653/cerm.2011.38.1.24 22384414 PMC3283046

[11] Gomis J, Cuello C, Sanchez-Osorio J, Gil MA, Parrilla I, Angel MA, et al Forskolin improves the cryosurvival of in vivo-derived porcine embryos at very early stages using two vitrification methods. Cryobiology. 2013;null. 10.1016/j.cryobiol.2012.12.009 23313786

[12] Kuleshova LL, MacFarlane DR, Trounson AO, Shaw JM. Sugars exert a major influence on the vitrification properties of ethylene glycol-based solutions and have low toxicity to embryos and oocytes. Cryobiology. 1999;38: 119–130. 10.1006/cryo.1999.2153 10191035

[13] Pedro PB, Zhu SE, Makino N, Sakurai T, Edashige K, Kasai M. Effects of hypotonic stress on the survival of mouse oocytes and embryos at various stages. Cryobiology. 1997;35: 150–158. 10.1006/cryo.1997.2034 9299106

[14] Shaw JM, Kuleshova LL, MacFarlane DR, Trounson AO. Vitrification properties of solutions of ethylene glycol in saline containing PVP, Ficoll, or dextran. Cryobiology. 1997;35: 219–229. 10.1006/cryo.1997.2043 9367610

[15] Wusteman MC, Pegg DE, Wang L-H, Robinson MP. Vitrification of ECV304 cell suspensions using solutions containing propane-1,2-diol and trehalose. Cryobiology. 2003;46: 135–145. 10.1016/S0011-2240(03)00019-1 12686203

[16] He X, Park EYH, Fowler A, Yarmush ML, Toner M. Vitrification by ultra-fast cooling at a low concentration of cryoprotectants in a quartz micro-capillary: a study using murine embryonic stem cells. Cryobiology. 2008;56: 223–232. 10.1016/j.cryobiol.2008.03.005 18462712 PMC2728604

[17] Vajta G, Holm P, Kuwayama M, Booth PJ, Jacobsen H, Greve T, et al Open Pulled Straw (OPS) vitrification: a new way to reduce cryoinjuries of bovine ova and embryos. Mol Reprod Dev. 1998;51: 53–58. 10.1002/(SICI)1098-2795(199809)51:1<53::AID-MRD6>3.0.CO;2-V 9712317

[18] Vajta G, Holm P, Greve T, Callesen H. Direct in-straw rehydration after thawing of vitrified in vitro produced bovine blastocysts. Vet Rec. 1995;137: 672 8966976

[19] Schiewe MC, Zozula S, Anderson RE, Fahy GM. Validation of microSecure vitrification (μS-VTF) for the effective cryopreservation of human embryos and oocytes. Cryobiology. 2015;71: 264–72. 10.1016/j.cryobiol.2015.07.009 26210008

[20] Kuwayama M, Vajta G, Kato O, Leibo SP. Highly efficient vitrification method for cryopreservation of human oocytes. Reprod Biomed Online. 2005;11: 300–8. 10.1016/s1472-6483(10)60837-1 16176668

[21] Santos MV, Sansinena M, Chirife J, Zaritzky N. Determination of heat transfer coefficients in plastic French straws plunged in liquid nitrogen. Cryobiology. Elsevier Inc.; 2014; 10.1016/j.cryobiol.2014.10.010 25445573

[22] Iwayama H, Hochi S, Kato M, Hirabayashi M, Kuwayama M, Ishikawa H, et al Effects of cryodevice type and donors’ sexual maturity on vitrification of minke whale (Balaenoptera bonaerensis) oocytes at germinal vesicle stage. Zygote. 2004;12: 333–338. 10.1017/s0967199404002928 15751543

[23] Liu Y, Du Y, Lin L, Li J, Kragh PM, Kuwayama M, et al Comparison of efficiency of open pulled straw (OPS) and Cryotop vitrification for cryopreservation of in vitro matured pig oocytes. Cryo Letters. 2008;29: 315–320. 19137194

[24] Bielanski A. A review of the risk of contamination of semen and embryos during cryopreservation and measures to limit cross-contamination during banking to prevent disease transmission in ET practices. Theriogenology. 2012;77: 467–482. 10.1016/j.theriogenology.2011.07.043 21958629

[25] Cobo A, Bellver J, de los Santos MJ, Remohí J. Viral screening of spent culture media and liquid nitrogen samples of oocytes and embryos from hepatitis B, hepatitis C, and human immunodeficiency virus chronically infected women undergoing in vitro fertilization cycles. Fertil Steril. 2012;97: 74–78. 10.1016/j.fertnstert.2011.10.006 22035968

[26] Criado E, Moalli F, Polentarutti N, Albani E, Morreale G, Menduni F, et al Experimental contamination assessment of a novel closed ultravitrification device. Fertil Steril. 2011;95: 1777–1779. 10.1016/j.fertnstert.2010.12.044 21269610

[27] Tedder RS, Zuckerman MA, Goldstone AH, Hawkins AE, Fielding A, Briggs EM, et al Hepatitis B transmission from contaminated cryopreservation tank. Lancet. 1995;346: 137–140. 10.1016/s0140-6736(95)91207-x 7603227

[28] Dubochet J. Cryo-EM—the first thirty years. J Microsc. 2012;245: 221–224. 10.1111/j.1365-2818.2011.03569.x 22457877

[29] Moor H. Recent progress in the freeze-etching technique. Philos Trans R Soc Lond B Biol Sci. 1971;261: 121–131. 10.1098/rstb.1971.0042 4399199

[30] Moor H, Bellin G, Sandri C, Akert K. The influence of high pressure freezing on mammalian nerve tissue. Cell Tissue Res. 1980;209: 201–216. 10.1007/BF00237626 6994890

[31] Gilkey JC, Staehelin LA. Advances in ultrarapid freezing for the preservation of cellular ultrastructure. J Electron Microsc Tech. 1986;3: 177–210. 10.1002/jemt.1060030206

[32] Studer D, Humbel BM, Chiquet M. Electron microscopy of high pressure frozen samples: bridging the gap between cellular ultrastructure and atomic resolution. Histochem Cell Biol. 2008;130: 877–889. 10.1007/s00418-008-0500-1 18795316

[33] Leunissen JL, Yi H. Self-pressurized rapid freezing (SPRF): a novel cryofixation method for specimen preparation in electron microscopy. J Microsc. 2009;235: 25–35. 10.1111/j.1365-2818.2009.03178.x 19566624

[34] Han H-MM, Huebinger J, Grabenbauer M. Self-pressurized rapid freezing (SPRF) as a simple fixation method for cryo-electron microscopy of vitreous sections. J Struct Biol. 2012/04/18. 2012;178: 84–87. 10.1016/j.jsb.2012.04.001 22508105

[35] Grabenbauer M, Han H-M, Huebinger J. Cryo-fixation by self-pressurized rapid freezing. Methods Mol Biol. 2014;1117: 173–91. 10.1007/978-1-62703-776-1_9 24357364

[36] Seki S, Mazur P. The dominance of warming rate over cooling rate in the survival of mouse oocytes subjected to a vitrification procedure. Cryobiology. 2009;59: 75–82. 10.1016/j.cryobiol.2009.04.012 19427303 PMC2729265

[37] Gualtieri R, Mollo V, Barbato V, Fiorentino I, Iaccarino M, Talevi R. Ultrastructure and intracellular calcium response during activation in vitrified and slow-frozen human oocytes. Hum Reprod. 2011;26: 2452–2460. 10.1093/humrep/der210 21715449

[38] Al-Amoudi A, Norlen LPO, Dubochet J. Cryo-electron microscopy of vitreous sections of native biological cells and tissues. J Struct Biol. 2004;148: 131–135. 10.1016/j.jsb.2004.03.010 15363793

[39] Bouchet-Marquis C, Starkuviene V, Grabenbauer M. Golgi apparatus studied in vitreous sections. J Microsc. 2008;230: 308–316. 10.1111/j.1365-2818.2008.01988.x 18445161

[40] Buser C, Walther P. Freeze-substitution: the addition of water to polar solvents enhances the retention of structure and acts at temperatures around -60 degrees C. J Microsc. 2008;230: 268–277. 10.1111/j.1365-2818.2008.01984.x 18445157

[41] Pierson J, Fernández JJ, Bos E, Amini S, Gnaegi H, Vos M, et al Improving the technique of vitreous cryo-sectioning for cryo-electron tomography: electrostatic charging for section attachment and implementation of an anti-contamination glove box. J Struct Biol. 2010;169: 219–225. 10.1016/j.jsb.2009.10.001 19822214

[42] Yakovlev S, Downing KH. Freezing in sealed capillaries for preparation of frozen hydrated sections. J Microsc. 2011;244: 235–47. 10.1111/j.1365-2818.2011.03575.x 22077543 PMC4199587

[43] Mazur P, Seki S. Survival of mouse oocytes after being cooled in a vitrification solution to -196°C at 95° to 70,000°C/min and warmed at 610° to 118,000°C/min: A new paradigm for cryopreservation by vitrification. Cryobiology. 2011;62: 1–7. 10.1016/j.cryobiol.2010.10.159 21055397 PMC3041861

[44] Miyata K, Hayakawa S, Kajiwara K, Kanno H. Supercooling and vitrification of aqueous glycerol solutions at normal and high pressures. Cryobiology. 2012;65: 113–116. 10.1016/j.cryobiol.2012.05.002 22609515

[45] Rubinsky B, Perez PA, Carlson ME. The thermodynamic principles of isochoric cryopreservation. Cryobiology. 2005;50: 121–38. 10.1016/j.cryobiol.2004.12.002 15843002

[46] Preciado JA, Rubinsky B. Isochoric preservation: a novel characterization method. Cryobiology. 2010;60: 23–9. 10.1016/j.cryobiol.2009.06.010 19559692

[47] Mikus H, Miller A, Nastase G, Serban A, Shapira M, Rubinsky B. The nematode Caenorhabditis elegans survives subfreezing temperatures in an isochoric system. Biochem Biophys Res Commun. 2016;477: 401–405. 10.1016/j.bbrc.2016.06.089 27329812

