# Supplementary figures and images for: Reversible Cryopreservation of Living Cells Using an Electron Microscopy Cryo-Fixation Method
Source: PLoS One. 2016 Oct 6;11(10):e0164270. doi: 10.1371/journal.pone.0164270 (PMC5053471; doi:10.1371/journal.pone.0164270)

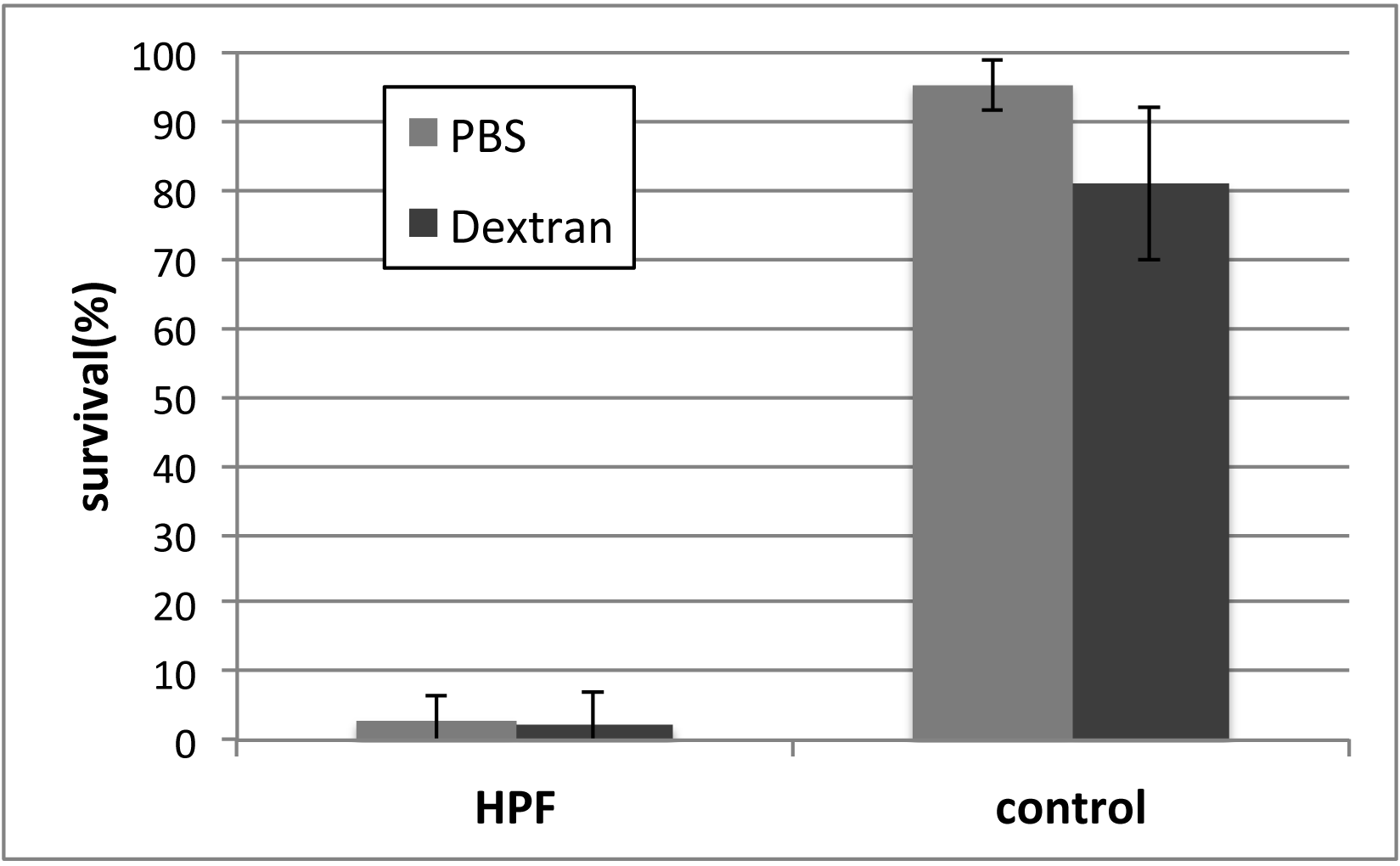

Supplement: S1 Fig — HeLa cells were subjected to high pressure freezing in either PBS or PBS supplemented with 30% dextran. They were subsequently thawed in 37°C warm cell culture medium and their viability was assessed by their ability to re-adhere after 6 h of culturing. As a control, unfrozen cells from the same samples were cultured. Data are represented as mean ± s.d.; n = 5–8. (TIF) [file pone.0164270.s001.tif]

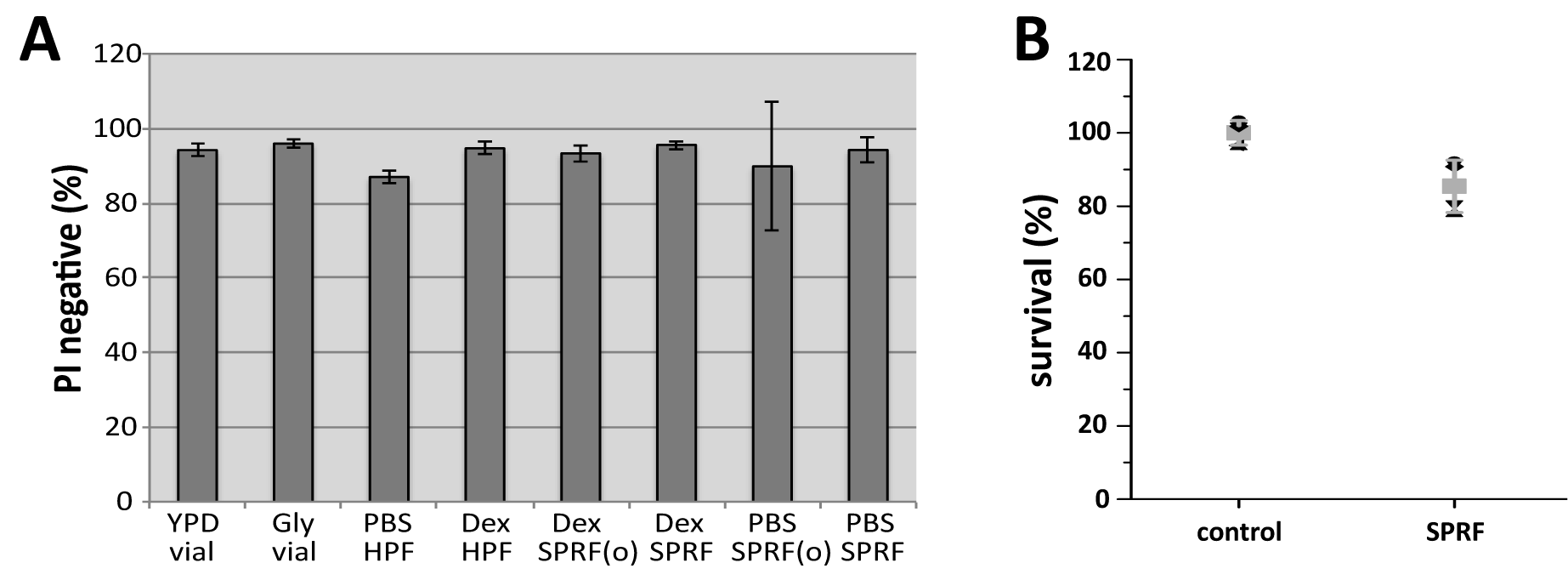

Supplement: S2 Fig — A: Viability of S. cerevisiae after different freezing procedures, quantified by PI staining. S. cerevisiae were diluted in YPD-medium, 15% glycerol in YPD (Gly), PBS or 30% dextran in PBS (Dex). They were subjected to conventional freezing in cryo-vials (vial), high-pressure freezing or self-pressurized rapid freezing (SPRF). After subsequent thawing integrity of yeast cells was evaluated by PI-staining. Data is represented as mean ± s.d.; n = 5. B: Viability of S. cerevisiae after SPRF in PBS without cryoprotection, quantified by measuring the optical density at 600 nm after 24 h of growth in YPD medium at 30°C and 200 rpm. Control: S. cerevisiae from corresponding samples filled in SPRF tubes but not frozen. Data is normalized to the corresponding controls. Black marks are single experiments; in gray mean ± s.d. are represented; n = 5 (TIF) [file pone.0164270.s002.tif]
